# Chronic hypoxia has differential effects on constitutive and antigen-stimulated immune function in Atlantic salmon (*Salmo salar*)

**DOI:** 10.3389/fimmu.2025.1545754

**Published:** 2025-02-19

**Authors:** Isis Rojas, Mariana M. M. de Mello, Fábio S. Zanuzzo, Rebeccah M. Sandrelli, Ellen de Fátima C. Peroni, Jennifer R. Hall, Matthew L. Rise, Elisabeth C. Urbinati, Anthony K. Gamperl

**Affiliations:** ^1^ Department of Ocean Sciences, Memorial University of Newfoundland, St. John’s, NL, Canada; ^2^ Aquaculture Center of Universidade Estadual Paulista (UNESP), São Paulo State University, Jaboticabal, São Paulo, Brazil; ^3^ Aquatic Research Cluster, Core Research Equipment & Instrument Training Network (CREAIT) Network, Ocean Sciences Centre, Memorial University of Newfoundland, St. John’s, NL, Canada

**Keywords:** hypoxia, immunity, climate change, aquaculture, *Aeromonas salmonicida*

## Abstract

Chronic hypoxia events are a common occurrence in Atlantic salmon (*Salmo salar*) sea-cages, especially during the summer, and their frequency and severity are predicted to increase with climate change. Although hypoxia is considered a very important fish health and welfare issue by the aquaculture industry, few studies have investigated the impact of chronic hypoxia on the fish immune system and its response to pathogen exposure. We exposed post-smolt Atlantic salmon to hypoxia (40% air sat.) for 6 weeks. Thereafter, we sampled fish prior to (i.e., at Time 0, to assess constitutive immune function), and after they were intraperitoneally injected with PBS (phosphate buffered saline) or formalin-killed *Aeromonas salmonicida*. We measured several innate immune parameters including: hematological immune responses [respiratory burst (RB), hemolytic activity of alternate complement system and plasma lysozyme concentration], and the relative percentage of circulating blood cells (erythrocytes/immature erythrocytes, monocytes, and granulocytes and lymphocytes) at Time 0 and at 24 hours post-injection (hpi); and the transcript expression levels of 8 anti-bacterial biomarkers in the head kidney [*interleukin-1 beta* (*il1b*), *interleukin-8a* (*il8a*), *cyclooxygenase-2* (*cox2*), *toll-like receptor 5, secreted* (*strl5*), *CC chemokine-like 19b* (*ccl19b*), *serum amyloid A5* (*saa5*), *hepcidin anti-microbial peptide a* (*hampa*) and *cathelicidin anti-microbial peptide b* (*campb*)] at Time 0 and at 6 and 24 hpi. In addition, we measured serum immunoglobulin (IgM) levels at Time 0 and at 8 weeks post-injection (4 weeks after a ‘boost’ injection). Fish exposed to chronic hypoxia had greater numbers of monocytes, which was consistent with the increase in RB, plasma lysozyme concentration and upregulated head kidney anti-bacterial gene expression (i.e., *campb*, *ccl19b*, *hampa*, *il8a*, *stlr5*). In contrast, chronic hypoxia: reduced RB and leukocyte numbers at 24 hpi in *Asal* compared to PBS-injected fish, and the transcript levels of *campb*, *il1b*, *saa5*, *il8a* and *stlr5* at 6- and/or 24- hpi; but had no effect on constitutive or post-stimulation serum IgM titers. Overall, our results indicate that chronic hypoxia has differential effects on salmon constitutive innate immune function vs. following antigen exposure, and thus, it is still unclear how chronic hypoxia will impact the capacity of fish to defend against pathogens.

## 1 Introduction

Atlantic salmon (*Salmo salar*) are farmed at coastal sea-cages sites, where they are vulnerable to changes in environmental conditions (1–3). For example, decreases in water oxygen levels (hypoxia) are a critical stressor (1, 4), and oxygen saturation levels of ≤ 60-70% are not that atypical in the summer (1, 5–7). Additionally, average sea surface temperatures have been increasing over the past several decades (8). This will also reduce water oxygen levels, and some models predict a further increase of 1.5-2°C in ocean temperatures as well as an increase in the incidence and severity of hypoxia (8, 9). Therefore, it is very likely that farmed Atlantic salmon will face lower levels of oxygen for longer periods if climate change continues unabated.

Hypoxia has been reported to induce a stress response that may modulate and/or compromise the innate immune system. Several studies have investigated the impacts of hypoxia on the fish immune system (10–16). However, most of these authors studied the effects of acute (short-term) or intermittent hypoxia (1, 16–26). Although, acute and intermittent hypoxia may also occur at salmon farms, only a few studies have examined the effects of prolonged exposure to low oxygen levels on fish immune function (12–15, 27–31). For instance, Martinez et al. (15) exposed Coho salmon (*Oncorhynchus kisutch*) to 100%, 60%, 50%, 35% and 25% air saturation (air sat.) for 28 days and reported increased toll-like receptor and cytokine transcript levels in fish held at oxygen levels below 60% air sat. In contrast, Leeuwis et al. (29) acclimated sablefish (*Anoplopoma fimbria*) for 16 weeks to normoxia (100% air sat.) and hypoxia (40% air sat.) at 10°C, and injected the fish with formalin-killed *Aeromonas salmonicida* (*Asal*) bacterin during the sixth and the tenth weeks. At the end of the experiment, whereas hypoxia had no/limited effects on the innate immune system, this condition prevented the increase in immunoglobulin (IgM) in those fish exposed to bacterin, suggesting that the adaptive immunity of the sablefish was compromised at low oxygen concentrations. Finally, Kvamme et al. (14) acclimated Atlantic salmon to hypoxia (52% air sat.) for 58 days and injected them with poly (I:C) or a *Vibrio* water-based vaccine, and reported that chronic hypoxia increased the stress response and reduced or delayed the expression of important immune-related transcripts in the head kidney. However, these results are difficult to interpret (i.e., what was the magnitude of immune suppression?) as the control group was exposed to water with an air sat. of only 74 ± 3.6%, and thus, these fish would already have been considered hypoxic (13).

Understanding how prolonged severe hypoxia affects the fish’s immune system, and the underlying mechanisms, is critical to predicting the impact of climate change on fish immune function and disease resistance, and may assist the aquaculture industry in developing strategies to mitigate production losses. Therefore, in this study, we exposed Atlantic salmon to 40% and 100% air sat. for 6 weeks before the injection of formalin-killed *Asal* or phosphate buffered saline (PBS), and measured a number of innate immune parameters prior to the injections (Time 0) and at 6 and 24 hours post-injection. These included: the respiratory burst (RB) of leukocytes, the hemolytic activity of the alternative pathway of the complement system, lysozyme concentration, the number of several types of leukocytes, and the transcript expression levels of 8 key immune-related genes in the head kidney. In addition, we measured serum IgM titers both pre-injection (at Time 0) and 8 weeks after PBS and *Asal* injection.

## 2 Materials and methods

The experimental procedures in this study were approved by the Institutional Animal Care Committee of Memorial University of Newfoundland (MUN; Protocol #17-94-KG) and performed in accordance with the guidelines of the Canadian Council on Animal Care (32).

### 2.1 Animals and experimental design

**Figure 1:**
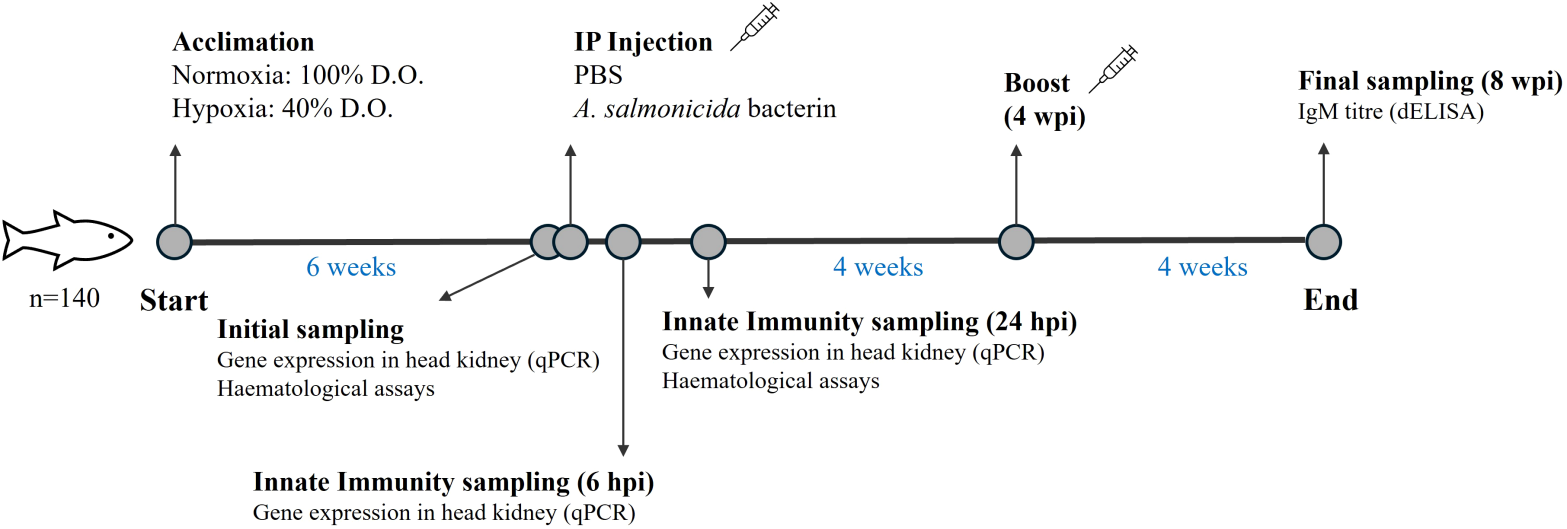
Schematic diagram showing the design of the experiment.

Atlantic salmon were obtained from Cape D’or Sustainable Seafood (Nova Scotia, Canada) and initially held at 10°C in seawater at the Dr. Joe Brown Aquatic Research Building (JBARB; Ocean Sciences Centre, MUN) in a 6,000 L circular tank for 1 month. During this period, the fish were fed with a commercial salmonid diet (Optiline MicroBalance, Skretting, St. Andrews, NB, Canada) at a ration of 1.5% biomass daily (body weight·day^−1^), and photoperiod was 12 h light:12 h dark. After acclimation, 140 fish were netted, anesthetized in seawater containing tricaine methanesulfonate (TMS; AquaLife TMS, 0.1 g·L^−1^; Syndel Laboratories Ltd, Nanaimo, BC, Canada), weighed, and had a PIT (Passive Integrated Transponder) tag implanted in their peritoneal cavity for identification. After tagging, the fish (117.4 ± 28.1 g) were randomly distributed in two 250 L tanks at the Cold-Ocean and Deep-Sea Research Facility (CDRF; Ocean Sciences Centre, MUN) to recover for 3 weeks before the experiment started. Thereafter, a WitroxCTRL Oxygen System (Loligo^®^ Systems, Viborg, Denmark) was used to monitor and reduce dissolved oxygen (DO) levels in one tank (hypoxia treatment) by 5% every two days until they reached 40% air saturation, this the lowest level of hypoxia that the salmon eat enough to maintain their body weight (33). After 6 weeks at these oxygen levels, 8 fish from both the normoxic (100% air sat.) and hypoxic tanks were sampled (initial sampling, Time 0) for blood and head kidney, and the remaining fish were anaesthetized (AquaLife TMS, 0.1 g·L^−1^) and intraperitoneally injected with 1 μL·g^-1^ of formalin-killed *Asal* or phosphate buffered saline (PBS) (29), and returned to their tanks. Seven (see Results) to eight fish per treatment were sampled at 6 and 24 hours post-injection (hpi; for both the PBS and *Asal* groups) for measures of innate immunity. Finally, four weeks after the initial injection, the remaining fish were again anaesthetized (AquaLife TMS, 0.1 g·L^−1^) and given a booster (1 μL·g^-1^ intraperitoneal injection of *Asal* or PBS). These fish were sampled for blood 4 weeks later [i.e. 14 weeks after the hypoxic fish reached 40% air sat. so that IgM titers could be measured (see below)]. During the experimental period, the water temperature was 10.3 ± 0.4°C, photoperiod was 12 h light:12 h dark and fish were fed at 1% body mass day^−1^. For a schematic diagram of the experimental protocol see **Figure 1**.

### 2.2 Bacteria preparation and injection

The *Asal* strain used in this study was isolated by Vasquez et al. (34). The bacteria were cultured in Trypticase Soy Broth (TSB) media (Difco, Franklin Lakes, NJ), and the obtained bacterial cell suspension was washed three times with PBS (pH 7.2, Gibco) by centrifugation at 4200×*g* for 10 min at 4°C. The *Asal* cells were then inactivated with 6% formaldehyde for 3 days at room temperature with gentle agitation. Formalin was removed by centrifugation at 4200×*g* for 10 min at 4°C and the cells were re-suspended in PBS. The bacterin cell concentrate was quantified using a Bacteria Counting Kit (Invitrogen, Thermo Fisher Scientific^®^, Mississauga, ON, Canada) for flow cytometry based on the manufacturers’ instructions. The bacterin was stored at 4°C at a concentration of 3.5 × 10^10^ CFU mL^-1^ until use, and then diluted in PBS to 10^8^ CFU mL^−1^ prior to injection. This is the same procedure used in (29), and thus allows us to directly compare the immune responses of the Atlantic salmon and sablefish to this bacterin formulation.

### 2.3 Sampling

At each sampling, fish were quickly euthanized in seawater containing TMS (Aqualife TMS, 0.4 g·L^−1^), measured and weighed. The blood was collected by puncture of the caudal vessels using a heparinized (1,000 IU·mL^-1^) and a non-heparinized (with 21-G needle) syringe, and the head kidney was removed using RNase AWAY^®^- (Cat. No. 83931, Sigma-Aldrich^®^, Oakville, ON, Canada) cleaned surgical instruments. The blood collected with the non-heparinized syringe was maintained in a refrigerator overnight and centrifuged the next morning for 10 min at 10,000×*g* to obtain serum. The serum was stored in 1.5 mL RNase-free tubes at -80°C prior to the determination of IgM concentration. An aliquot of blood collected with a heparinized syringe at 24 hpi was immediately used in the leukocyte respiratory burst (RB) activity assay, and another aliquot was used for blood cell counting using flow cytometry. The remaining blood was centrifuged for 1 min at 10,000×*g* to separate the plasma. The plasma was then distributed into different 1.5 mL RNase-free tubes, immediately frozen in liquid nitrogen, and stored at -80°C prior to the determination of lysozyme concentration and the hemolytic activity of the alternative pathway of the complement system. At 6 and 24 hpi, a portion of the head kidney was sampled and placed into 1.5 mL RNase-free tubes, snap-frozen in liquid nitrogen, and stored at -80°C until preparation of RNA for immune-related transcript expression analyses.

### 2.4 Innate immune indicators

Innate humoral immune parameters were measured on initial (Time 0) samples and those collected 24 hpi in PBS- and formalin-killed *Asal*-injected fish to evaluate the effect of chronic hypoxia, and PBS vs. *Asal* injection, on innate immune indicators.

#### 2.4.1 Respiratory activity of leukocytes

The production of reactive oxygen species by salmon blood leukocytes was measured using chemiluminescence following the methods described by Marnila et al. (35) and Nikoskelainen (36). Briefly, whole blood (2 µL·mL^-1^) was added to a mixture of 1 mM luminol (5-amino-2,3-dihydro-1,4-phthalazinedione, Cat. No. 820071, Sigma-Aldrich^®^) in 0.2 M sodium borate and 1X PBS. Then, 225 µL of this solution was added to triplicate wells of a 96-well white cliniplate (Cat. No. 28298-610, VWR International^®^, Mississauga, ON, Canada) followed by 25 µL of 20 mg·mL^-1^ zymosan from *Saccharomyces cerevisiae* (Cat. No. Z4250, Sigma-Aldrich^®^). Finally, chemiluminescence was measured over 60 min using a microplate reader (SpectraMax M5; Molecular Devices, Sunnydale, CA, USA) at room temperature. From this, a curve of luminescence counts per second (LCS) was obtained, and peak values were taken to represent the respiratory burst of each sample.

#### 2.4.2 Hemolytic activity of the alternative pathway of the complement system

This parameter was determined according to Zanuzzo et al. (37). A serum sample was diluted (1:8) in TEA-EGTA-Mg^2+^ buffer (triethanolamine ethylene glycol tetraacetic acid; 8 mM, with 2 mM of Mg^2+^ and 0.1% gelatin, pH 7.4), and used to resuspend rabbit erythrocytes (RaRBC) in TEA-EGTA/Mg^2+^/gelatin 0.1%. A kinetic assay was then used to determine the time (in seconds) necessary for the serum sample to lyse 50% of the RaRBC suspension by measuring the absorbance of the sample at 700 nm every 20 sec for 20 min.

#### 2.4.3 Lysozyme concentration

Plasma lysozyme concentration was determined according to Zanuzzo et al. (37) and Demers et al. (38) based on lysis of a *Micrococcus lysodeikticus* suspension (Cat. No. M3770, Sigma^®^). The samples were incubated for 30 min at 40°C to inactivate complement proteins, and then added to the *M. lysodeikticus* suspension. The reduction in absorbance of this solution was measured at 450 nm for 10 min using a plate reader.

### 2.5 Blood cell counts

The counting of blood cells was performed on Time 0 and 24 hpi PBS- and formalin-killed *Asal*-injected fish, following the protocol described in Inoue et al. (39). A stock solution of 3,3-dihexyloxacarbocyanine (DiOC_6,_ Cat. No. D273, ThermoFisher Scientific^®^) was diluted using 1:10 Hanks balanced salt solution (HBSS) and added to a whole blood aliquot diluted in deionized water (1:19). The solution was filtered through a 100 μM mesh, centrifuged for 5 min at 500×*g*, and the obtained pellet was resuspended in Fluorescence-activated cell sorting (FACs) buffer. The samples were read using a BD FACSCalibur^®^ Flow Cytometer (Becton Dickinson, San Jose, CA, USA) using BD FACSDiva software, which identifies each blood cell population by its location in a Forward scatter (FSC) vs. Side scatter (SSC) plot (see **Figure 3E**).

### 2.6 Immune-related gene expression analyses

To further evaluate the effects of chronic hypoxia and bacterial antigen administration on fish immunity, we selected 8 genes (cathelicidin anti-microbial peptide b – *campb*, CC chemokine-like 19b – *ccl19b*, cyclooxygenase-2 – *cox2*, hepcidin anti-microbial peptide a – *hampa*, interleukin-1 beta – il1b, interleukin-8a – *il8a*, serum amyloid A5 – *saa5* and toll-like receptor 5, secreted – *strl5*) based on their functional activity, and these include immune receptors, cytokines, antimicrobial effectors and inflammatory mediators (40–46). Transcript expression levels of these genes in head kidney samples from the normoxia- and hypoxia-acclimated fish pre-injection, and at 6 and 24 hpi with either PBS or formalin-killed *Asal*, were assessed using real-time quantitative polymerase chain reaction (qPCR) analyses. All of the primer pairs utilized in the qPCR analyses conducted herein have been previously reported and subjected to quality assurance testing (see **Table 1** for detailed primer information, including references).

**Table 1:** qPCR primer sequences used to examine the effects of chronic hypoxia on the immune response of Atlantic salmon.

Gene name (symbol) (GenBank Acc. No.)	Nucleotide sequence (5’-3’)	Efficiency (%)	Amplicon size (bp)	Reference
cathelicidin anti-microbial peptide b (*campb*) (AY360357)	F: AGACTGGCAACACCCTCAAC	102	112	(42)
	R: TTGCCTCTTCTTGTCCGAAT			
CC chemokine-like 19b (*ccl19b*) (BT058161)	F: CTGCTTGACAACGACCGATA	90	151	(40)
	R: GTTGTTCTTGGTGGCAGGAG			
cyclooxygenase-2 (*cox2*) (AY848944)	F: ACCTTTGTGCGAAACGCTAT	105	113	(40)
	R: GAGTAGGCCTCCCAGCTCTT			
hepcidin anti-microbial peptide a (*hampa*) (BT125319)	F: ATGAATCTGCCGATGCATTTC	96	134	(42)
	R: AATGGCTTTAGTGCTGGCAG			
interleukin-1 beta (*il1b*) (AY617117)	F: GTATCCCATCACCCCATCAC	97	119	(44)
	R: TTGAGCAGGTCCTTGTCCTT			
interleukin-8a (*il8a*) (BT046706)	F: GAAAGCAGACGAATTGGTAGAC	103	99	(44)
	R: GCTGTTGCTCAGAGTTGCAAT			
serum amyloid A5 (*saa5*) (BT057477)	F: AGGAGCTGGAAGTTTGTTGC	101	143	(46)
	R: TATGCACGCCACATGTCTTT			
toll-like receptor 5, secreted (*stlr5*) (AY628755)	F: ATCGCCCTGCAGATTTTATG	105	103	(45)
	R: GAGCCCTCAGCGAGTTAAAG			
^a,b^60S ribosomal protein L32 (*rpl32*) (BT043656)	F: AGGCGGTTTAAGGGTCAGAT	100	119	(47)
	R: TCGAGCTCCTTGATGTTGTG			
^a^β-actin (*actb*) (BG933897)	F: CCAAAGCCAACAGGGAGAAG	100	91	(47)
	R: AGGGACAACACTGCCTGGAT			
^a^elongation factor 1-alpha 1 (*ef1a1*) (AF321836)	F: TGGCACTTTCACTGCTCAAG	102	197	(48)
	R: CAACAATAGCAGCGTCTCCA			
^a^elongation factor 1-alpha 2 (*ef1a2*) (BT058669)	F: GCACAGTAACACCGAAACGA	98	132	(49)
	R: ATGCCTCCGCACTTGTAGAT			
^a^eukaryotic translation initiation factor 3 subunit D (*eif3d*) (GE777139)	F: CTCCTCCTCCTCGTCCTCTT	106	105	(40)
	R: GACCCCAACAAGCAAGTGAT			
^a,b^polyadenylate-binding protein cytoplasmic 1 (*pabpc1*) (EG908498)	F: TGACCGTCTCGGGTTTTTAG	102	108	(50)
	R: CCAAGGTGGATGAAGCTGTT			

a Candidate normalizers.

b Expression levels of the transcripts of interest (TOIs) were normalized to transcript expression levels of these two genes.

Head kidney samples (approximately 100 mg of tissue) were homogenized in 400 µL of TRIzol Reagent (Cat. No. 15596-018, Invitrogen/ThermoFisher Scientific^®^) using a motorized Kontes RNase-Free Pellet Pestle Grinder (Kimble Chase, Vineland, NJ, USA). An additional 400 µL of TRIzol Reagent (Invitrogen/ThermoFisher Scientific^®^) were then added, mixed by pipetting, and the homogenates were frozen in dry ice and stored at -80°C. Frozen homogenates were further processed by slowly thawing them on ice, and then passing them through a QIAshredder (QIAGEN, Mississauga, ON, Canada) spin column following the manufacturer’s instructions. Two-hundred µL of TRIzol (Invitrogen/ThermoFisher Scientific^®^) were then added to each sample to make a total homogenate volume of approximately 1 mL, and the TRIzol total RNA extractions were then completed following the manufacturer’s instructions. Total RNA samples (45 µg) were treated with 6.8 Kunitz units of DNaseI (RNase-Free DNase Set, Cat. No. 79254, QIAGEN) with the manufacturer’s buffer (1X final concentration) at room temperature for 10 min to degrade any residual genomic DNA. DNase-treated RNA samples were column-purified using the RNeasy Mini Kit (Cat. No. 74106, QIAGEN) following the manufacturer’s instructions. RNA integrity was verified by 1% agarose gel electrophoresis, and RNA purity was assessed using A260/280 and A260/230 ratios determined using a NanoDrop™ UV-Vis spectrophotometer (Thermo Scientific) for both the pre-cleaned and the column-purified RNA samples. Column-purified RNA samples had A260/280 ratios between 2.0 and 2.3 and A260/230 ratios between 1.9 and 2.3.

First-strand cDNA templates for qPCR were synthesized in 20 μL reactions from 1 µg of DNaseI-treated, column-purified, total RNA using random primers (250 ng; Cat. No. 48190-011, Invitrogen/Thermo Fisher Scientific^®^), dNTPs (0.5 mM final concentration; Cat. No. 10297-018, Invitrogen/Thermo Fisher Scientific^®^) and M-MLV reverse transcriptase (200 U; Cat. No. 28025-013, Invitrogen/Thermo Fisher Scientific^®^) with the manufacturer’s first strand buffer (1X final concentration) and DTT (10 mM final concentration) at 37°C for 50 min.

All PCR amplifications were performed in 13 µL reactions using 1X Power SYBR Green PCR Master Mix (Cat. No. A25776, Applied Biosystems/ThermoFisher Scientific^®^, Waltham, MA, USA), 50 nM of both the forward and reverse primers, and cDNA representing 4 ng of input total RNA. Amplifications were performed using the ViiA 7 Real Time PCR system (384-well format) (Applied Biosystems/Thermo Fisher Scientific^®^). The real-time analysis program consisted of 1 cycle of 50°C for 2 min, 1 cycle of 95°C for 10 min, and 40 cycles of 95°C for 15 sec and 60°C for 1 min, with fluorescence detection at the end of each 60°C step, and was followed by dissociation curve analysis.

Expression levels of the transcripts of interest (TOIs) were normalized to transcript levels of two endogenous controls. These endogenous controls were selected from six candidate normalizers [60S ribosomal protein L32 (*rpl32*), β-actin (*actb*), elongation factor 1-alpha 1 (*ef1a1*), elongation factor 1-alpha 2 (*ef1a2*), eukaryotic translation initiation factor 3 subunit D (*eif3d*) and polyadenylate-binding protein cytoplasmic 1 (*pabpc1*)]. Briefly, the fluorescence threshold cycle (C_T_) values of 30 samples (i.e. 3 samples from each of the 10 groups) were measured (in triplicate) for each of these transcripts, and then analyzed using geNorm (51). Based on this analysis, *rpl32* (geNorm M = 0.129) and *pabpc1* (geNorm M = 0.131) were selected as the two endogenous controls.

The qPCR analyses of expression levels of the 8 TOIs in the 78 head kidney samples were then performed using the ViiA 7 Real Time PCR system (384-well format) (Applied Biosystems/ThermoFisher Scientific^®^). On each plate, for every sample, the TOIs and endogenous controls were tested in triplicate, and a plate linker sample (i.e., a sample that was run on all plates in each study) and a no-template control were included. The relative quantity (RQ) of each transcript was determined using the ViiA 7 Software Relative Quantification Study Application (Version 1.2.3) (Applied Biosystems/ThermoFisher Scientific^®^), with normalization to both *rpl32* and *pabpc1* transcript levels, and with amplification efficiencies incorporated. For each TOI, the sample with the lowest normalized expression (mRNA) level was set as the calibrator sample (i.e., assigned an RQ value = 1.0).

### 2.7 Adaptative immunity

#### 2.7.1 IgM concentration

Salmon IgM purification was performed according to Vasquez et al. (34) with minor modifications. IgM was purified from pooled serum (~100 mL) collected from ~2 kg salmon, using an immobilized mannan binding protein (MBP) column kit (Cat. No. 44897, Pierce™, Thermo Scientific, USA) according to the manufacturer’s instructions. The integrity and purity of IgM in the elution fractions were evaluated by 10% SDS-PAGE (52) against a marker (PageRuler Plus Prestained Protein Ladder, Thermo Scientific) followed by Coomassie staining, while the concentration was estimated with DirectUV (absorbance at 280 nm) using a Genova-Nano spectrophotometer (Jenway, UK) and a bicinchoninic acid (BCA) assay (BCA protein assay kit, Pierce™, Thermo Scientific) using a plate reader (SpectraMax M5). High quality IgM fractions were pooled and dialyzed twice against 20 mM Tris buffer (pH 8.0) at 4°C with gentle stirring using a dialysis cassette or dialysis bag (3-20 kDa molecular weight cut-off, Thermo Scientific). Subsequently, the purified salmon IgM was lyophilized (EdwarDS-Super Modulyo, Boc Ltd, UK), re-suspended in 20 mM Tris buffer (pH 8.0), and re-evaluated for integrity and concentration using 10% SDS-PAGE and DirectUV. Finally, salmon IgM was stored at -20°C in aliquots to protect against repeated freeze-thaw cycles.

The production of chicken anti-salmon-IgM IgY was done commercially at Somru BioScience Inc. (Charlottetown, PEI, Canada), and involved affinity purification of the antibodies from chicken eggs using the antigen, followed by biotinylation of the antibodies.

We measured total IgM titers using dELISA according to Vasquez et al. (34) with some modifications. Serum samples were initially diluted 100,000x in carbonate-bicarbonate coating buffer (15 mM Na_2_CO_3_; 35 mM NaHCO_3_; pH 9.8) and heat-treated at 45°C for 30 min to inactivate complement activity. Then, 100 µL of each sample was pipetted into triplicate wells of high protein-binding polystyrene 96-well plates (Nunc MaxiSorp™, Thermo Fisher Scientific). For each plate, a pool of samples was diluted a further 30x in carbonate-bicarbonate coating buffer, and then added into duplicate wells (final dilution of 3,000,000x). The optical density (OD) of the pool was used to account for the non-specific background signal, and this value was deduced from each sample’s OD. Each plate included a standard curve with purified salmon IgM diluted in coating buffer to 3, 1.5, 0.75, 0.375, 0.1875, 0.0937 and 0.04687 µg·mL^-1^, and a negative control with only coating buffer, in duplicate wells. The plates were incubated at 4°C overnight and washed 4 times with 200 μL of 0.1% v/v Tween 20 in 1x PBS (PBS-T 0.1%) using a microplate washer (BioTek™ 50TS, Thermo Fisher Scientific) on the next day. Subsequently, the plates were blocked by adding 150 µL of ChonBlock™ (Cat. No. 9068, Chondrex Inc. WA, USA) in each well for 1 h at 37°C, followed by washing according to the procedure described previously. Then, plates were incubated with 100 μL of chicken anti-salmon-IgM IgY per well (diluted 1,000x in PBS-T 0.05%) for 1 h at 37°C. After washing, 100 μL of streptavidin-horseradish peroxidase (Cat. No. 405210, BioLegend, CA, USA; diluted 500X in PBS-T 0.05%) was added to each well, and the plates were incubated for 1 h at 37°C. Following washing, the plates were incubated with 100 μL of TMB solution (Kementec Solutions Inc. 4850, NH, USA) per well for 30 min at RT in the dark. The color/reaction was stopped by adding 100 μL of 0.3 M H_2_SO_4_. The bottom of the plates was wiped, and the plate read at 450 nm (SpectraMax M5; Molecular Devices, CA, USA). Based on the OD of the standards, serum IgM concentration was calculated using a 4-parameter sigmoidal regression using SoftMax Pro software.

### 2.8 Statistical analyses

All the data are reported as means ± 1 standard error of the mean (n=7 - 8), and P < 0.05 was used as the level of statistical significance. The data were analyzed using RStudio v. 2024.09.0 + 375 and R CRAN v. 4.4.1 (R Core Team, 2024, https://www.r-project.org/). All graphs were made using GraphPad Prism^©^ v. 10.0.0 for Windows (MA, USA). The interquartile range method was applied to identify outliers. Based on this analysis, 3 samples were removed: one fish in the hypoxia *Asal* 24 hpi group; one fish in the normoxia *Asal* 6 hpi group; and one fish in the normoxia *Asal* 24 hpi group. The data were inverse or log_2_ transformed if necessary to meet the ANOVA normality (Shapiro-Wilk test) and homoscedasticity (Fligner test) assumptions. If not, Kruskal-Wallis tests were performed, and Dunn’s *post-hoc* tests [package ‘*dunn.test’* v. *1.3.6* (53)] were used to identify differences between groups.

We performed two different analyses of variance (ANOVAs) as follows: a 2 (hypoxia and normoxia) x 2 (PBS and *Asal*) factorial test to examine the innate immune responses, the cellular blood count data and serum IgM levels; and a 2 (hypoxia and normoxia) x 2 (PBS and *Asal*) x 2 (6 and 24 hpi) factorial test to analyze gene expression. Both of these analyses were followed by Tukey’s [package ‘*agricolae’* v. *1.3-7* (54)] *post-hoc* tests to enable pairwise comparisons. To compare the basal (constitutive) gene expression between treatments, a t-test was used. Dunnett’s test [package ‘*FSA’* v. *0. 95* (55)] was performed to examine if values were different from basal values within each oxygen condition (treatment).

## 3 Results

In this study, we evaluated the effects of 6+ weeks of severe hypoxia (40% air sat.) on the constitutive (basal) and antigen-stimulated innate and acquired immune function of Atlantic salmon. However, this level of hypoxia had a number of other effects on the salmon. Salmon maintained under hypoxia were less active, spent time at the water’s surface, had an increased gill ventilation rate, and grew much slower than fish held in normoxic water (de Mello et al., unpubl). Two fish from the hypoxia treatment died after injection with formalin-killed *A. salmonicida*, however, these fish were without any external signs of disease/ill health.

### 3.1 Innate immune response

**Figure 2:**
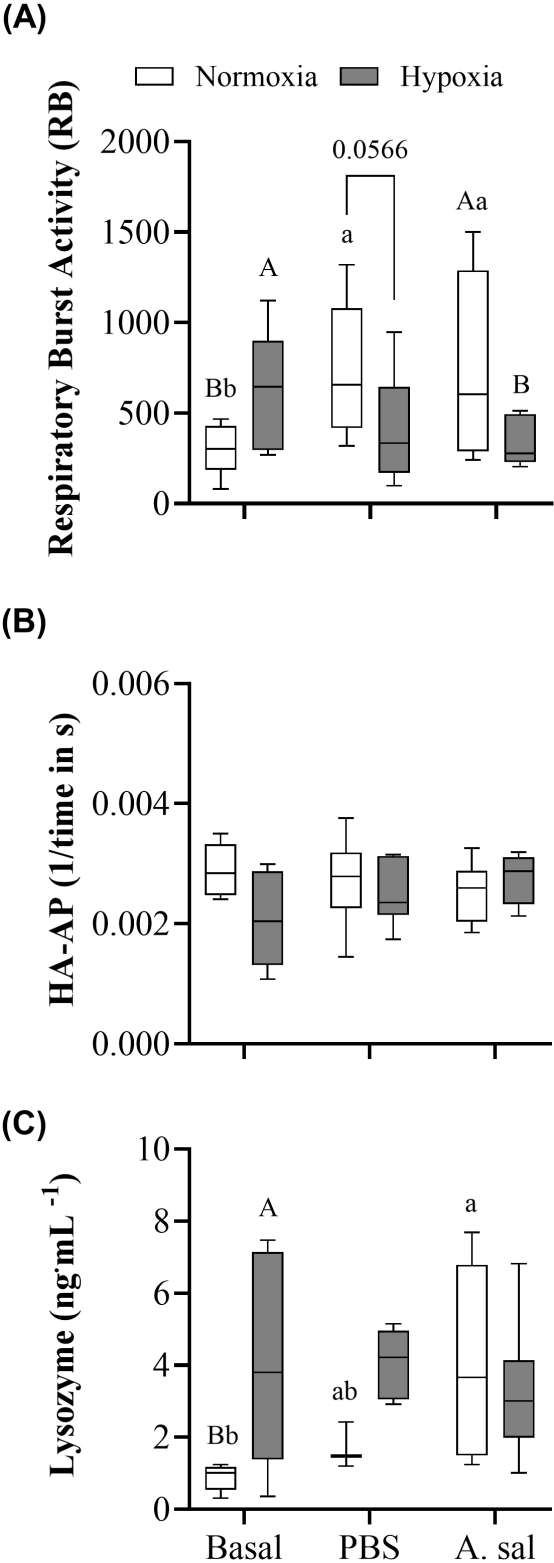
Blood respiratory burst activity (RB) of leukocytes **(A)**, hemolytic activity of the alternative pathway of the complement system (HAAP); **(B)** and plasma lysozyme concentration **(C)** in Atlantic salmon after 6 weeks of normoxia or hypoxia (40% air saturation), and 24 hours after these groups were injected with PBS or formalin-killed *A. salmonicida*. Different capital letters indicate a significant (P < 0.05) difference between treatments at the same sampling time. Different lowercase letters indicate differences between sampling times within the same treatment. Symbols were omitted when there was no statistically significant difference. Values are presented as means ± 1 standard error (n=7-8).

Constitutive (basal) leukocyte respiratory burst activity (RB) and lysozyme concentration were significantly greater (by 52 and 78%, respectively) in hypoxia-exposed vs. normoxic fish (**Figure 2**; **Supplementary Table S1**). At 24 hpi with PBS or attenuated *Asal* there was an approximately 2.5-fold increase in RB in normoxia-acclimated fish (**Figure 2A**). However, there were no significant differences between the initial sampling and both PBS and *Asal*-injected values for fish held at 40% air saturation. This difference in responsiveness resulted in RB in normoxia-acclimated fish being nearly significantly (P = 0.057) or significantly higher at 24 hpi in normoxia- vs. hypoxia-acclimated fish following PBS and *Asal* injection, respectively. A similar pattern was observed for lysozyme concentration (**Figure 2C**), where *Asal* injection into normoxia-acclimated, but not hypoxia-acclimated, fish resulted in an increase in this parameter as compared to initial values. However, in this case, the differences in PBS and *Asal* injected fish did not reach significance. No significant differences in initial or post-injection hemolytic activity of the complement system were found (**Figure 2B**).

### 3.2 Blood cells counts

**Figure 3:**
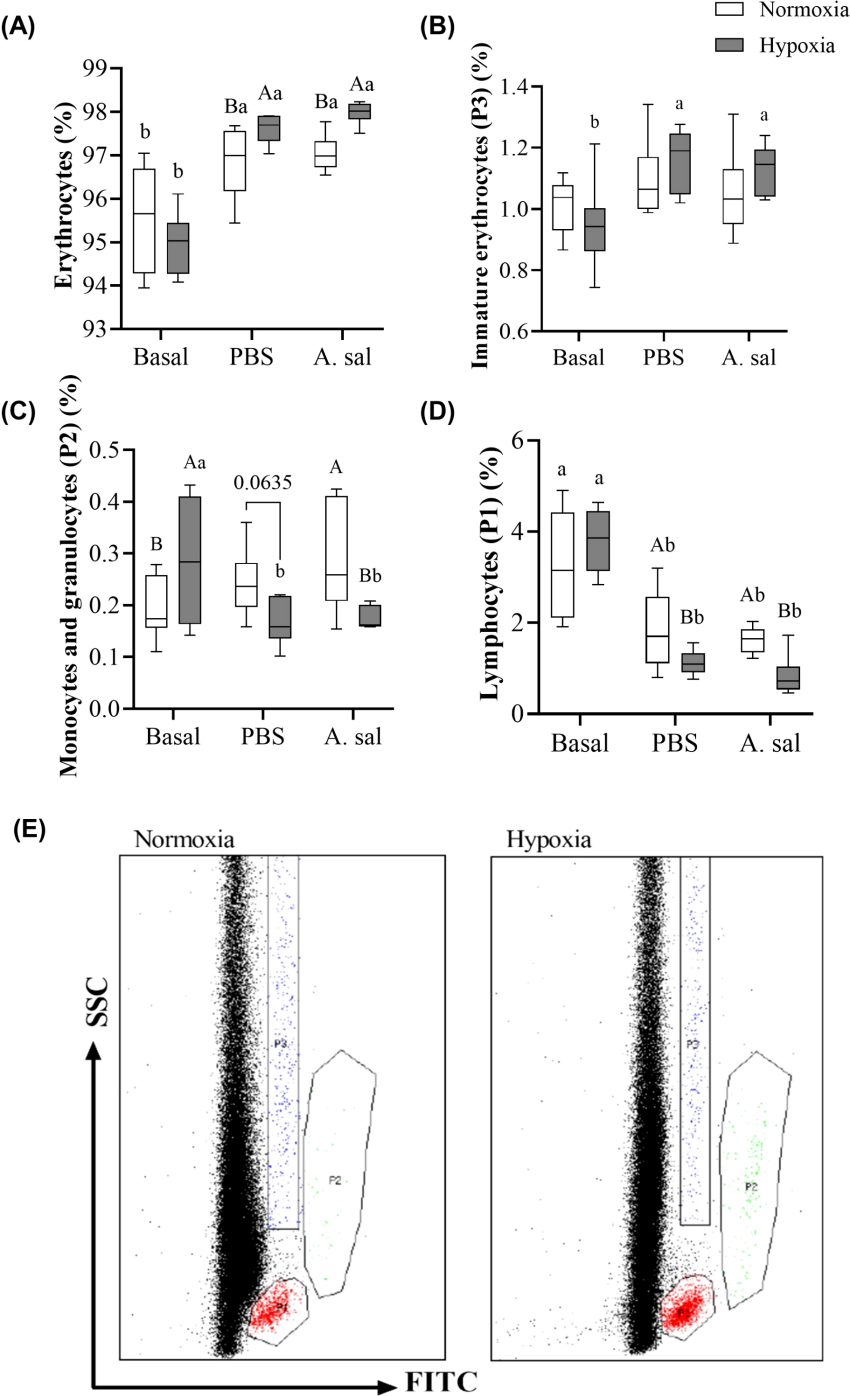
Percentage of erythrocytes **(A)**, immature erythrocytes **(B)**, monocytes and granulocytes **(C)** and lymphocytes **(D)** in Atlantic salmon after 6 weeks of hypoxia (40% air saturation) or normoxia, and 24 hours after these groups were injected with PBS or formalin-killed *A. salmonicida*. Different capital letters indicate a difference (P < 0.05) between treatments at the same sampling time. Different lowercase letters indicate differences between sampling times within the same treatment. Symbols were omitted when there was no statistically significant difference. Values are presented as means ± 1 standard error (n=7-8). Image **(E)** shows the different populations of cells obtained through flow cytometry in samples from normoxia- vs. hypoxia-acclimated fish.

The percentage of circulating monocytes and granulocytes decreased significantly after the injection of PBS or *Asal* in hypoxia-acclimated, but not normoxia-acclimated, fish compared to initial values (**Figure 3C**; **Supplementary Table S1**). This resulted in this parameter being lower (by 0.1 to 0.15%) in hypoxia- vs. normoxia-acclimated fish in the PBS-injected group at 24 hpi. The percentage of lymphocytes was not different pre-injection. The percentage of these cells fell in both groups following PBS and *Asa*l injection. However, the decrease was greater in hypoxia-acclimated fish, and this resulted in lower values (by approx. 1%) in both injection groups at 24 hpi.

Hypoxia-acclimation had no effect on the initial percentage of erythrocytes, however, this parameter was slightly higher in hypoxia-acclimated fish independent of whether they were injected with PBS or *Asal* (**Figure 3A**; **Supplementary Table S1**). Circulating immature erythrocytes increased in hypoxia-acclimated fish at 24 hpi when given either injection. However, these values were not significantly different from those measured in normoxia-acclimated fish at this time point (**Figure 3B**; **Supplementary Table S1**).

### 3.3 Gene expression

**Figure 4:**
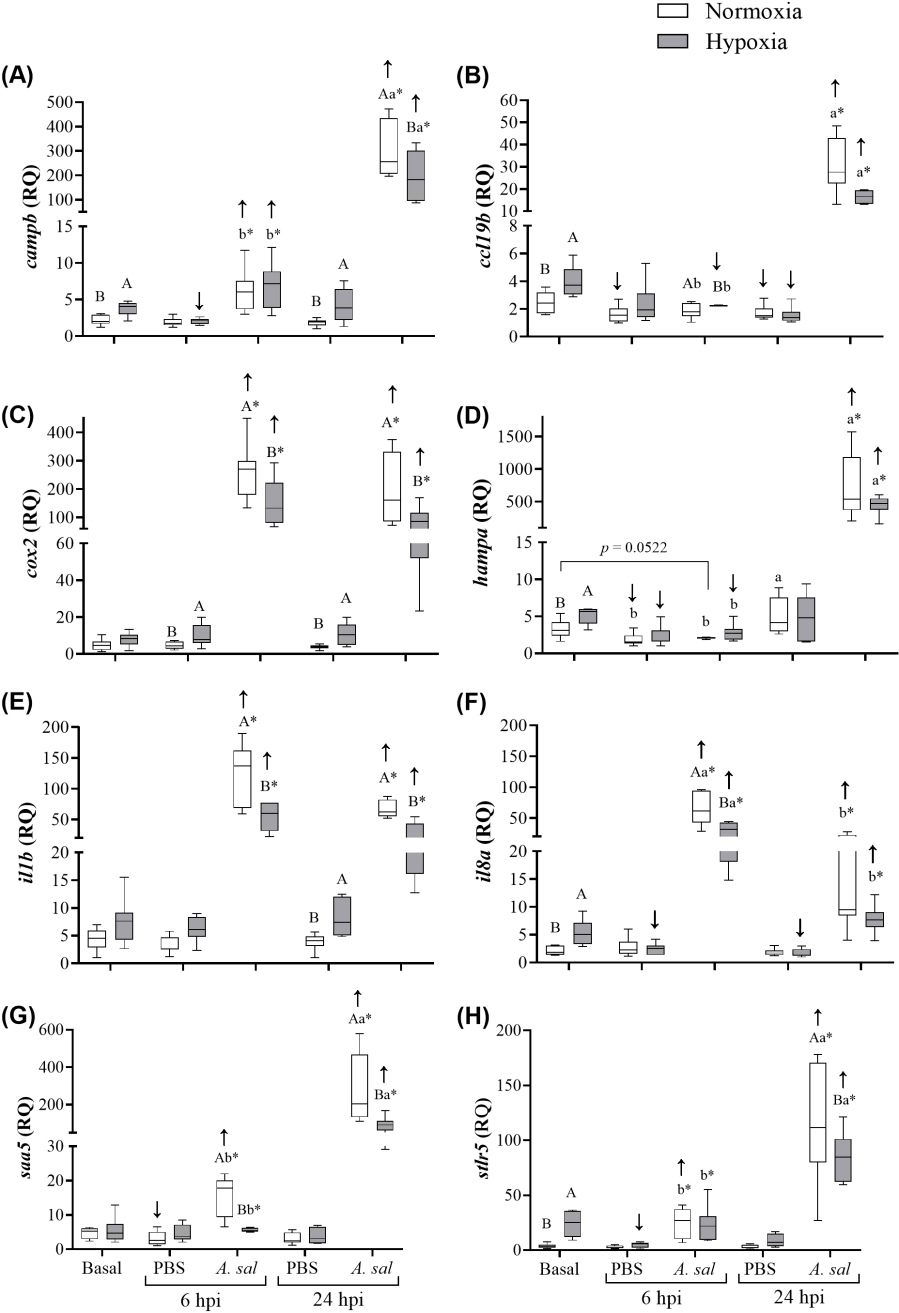
Transcript (mRNA) expression of 8 antibacterial biomarker genes in Atlantic salmon head kidney after 6 weeks of hypoxia (40% air saturation) or normoxia, and in these fish 6 and 24 hours post-injection (hpi) with PBS or formalin-killed *A. salmonicida*. **(A)** *campb*, **(B)**, *ccl19b*, **(C)** *cox2*, **(D)** *hampa*, **(E)** *il1b*, **(F)** *il8a*, **(G)** *saa5*, **(H)** *stlr5*. Different capital letters indicate significant (P < 0.05) differences between treatments at the same sampling time. Different lowercase letters indicate differences between sampling times (6 and 24 hpi) within the same treatment. Asterisks (*) indicate a difference between injection groups (PBS and *A. salmonicida*). Arrows indicate difference between initial sampling and each injection group at each sampling time. Symbols were omitted when there was no statistical difference. Values are presented as means ± 1 standard error (n=7-8).

Transcript expression levels of *campb*, *ccl19b*, *hampa*, *il8a* and *stlr5* were significantly higher (by 1.6-, 1.6-, 1.5-, 2.5- and 7-fold, respectively) at the initial sampling in the hypoxia- compared to normoxia-acclimated fish (**Figures 4A, B, D, F, H**, respectively; **Supplementary Table S2**). However, no significant differences were observed in *cox2*, *il1b* or *saa5* expression levels (**Figures 4C, E, G**, respectively; **Supplementary Table S2**).

In hypoxia-acclimated fish injected with PBS at 6 hpi, levels of *campb*, *hampa*, *il8a* and *stlr5* were significantly lower (by 45, 53, 54 and 81%, respectively) compared to basal expression levels. In contrast, in the normoxia-acclimated fish injected with PBS, levels of *ccl19b*, *hampa* and *saa5* were significantly lower (by 45, 42 and 17%, respectively) compared to basal expression levels. With the exception of *ccl19b* and *il8a* for the hypoxic fish, these values were not significantly lower at 24 hpi, and several genes (i.e., *campb*, *cox2*, *il1b*) were again more highly expressed in hypoxia- than normoxia-acclimated fish.

Injection of *Asal* into normoxia-acclimated Atlantic salmon substantially increased the expression of 6 of the 8 genes at 6 hpi (*ccl19b* and *hampa* were the exception), and all of the genes at 24 hpi. These changes ranged from 2.7- to 53-fold. A similar pattern of gene expression was observed in hypoxia-acclimated fish at the two post-injection sampling points. However, in hypoxia- compared to normoxia-acclimated fish, the magnitude of the increase in transcript expression (by 1.8- to 7.1-fold) was significantly lower at both sampling time points for *cox2*, *il1b* and *saa5*, and at 6 hpi or 24 hpi for *il8a* and *campb/stlr*, respectively, and a similar (but not statistically-significant) decrease was evident for *ccl19b* and *hampa* (53 and 62%, respectively) at 24 hpi.

### 3.4 Adaptive immune response

**Figure 5:**
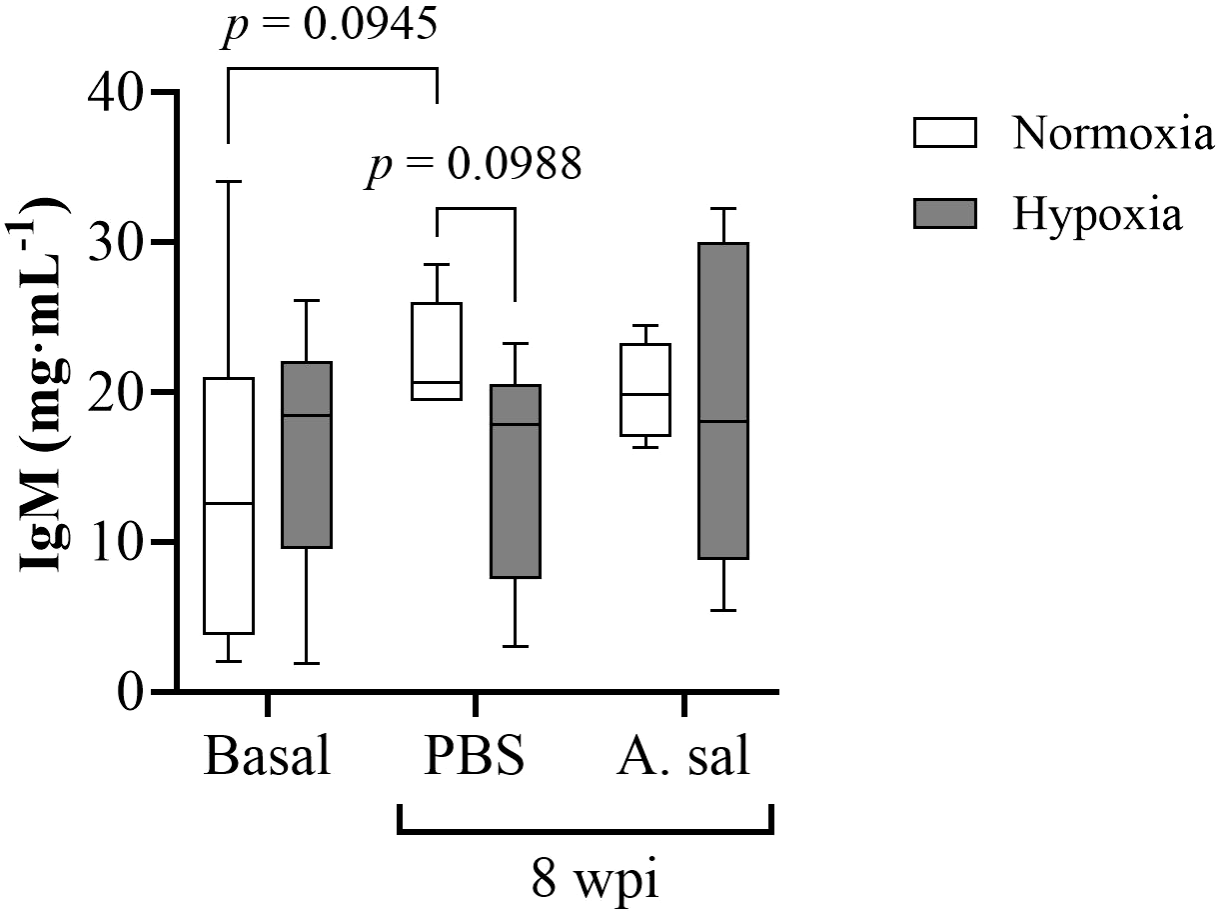
Serum IgM concentration in Atlantic salmon after 6 weeks of hypoxia (40% air saturation) or normoxia, and 8 weeks postinjection (wpi) with PBS or formalin-killed *A. salmonicida*. Values are presented as means ± 1 standard error (n=7-8).

Despite serum IgM levels being 39% higher in the normoxia-acclimated fish after the PBS injection compared to the initial sampling, and 33% higher than in the hypoxia-acclimated fish following PBS injection (P < 0.10), no significant differences were found in constitutive IgM levels between the normoxia- and hypoxia-acclimated groups, or for either injection group as compared to pre-injection (initial) values (**Figure 5**; **Supplementary Table S1**).

## 4 Discussion

### 4.1 Effect of chronic hypoxia on basal immune function

#### 4.1.1 Humoral immunity and blood cell populations

The innate, or non-specific, immune system provides the first line of defense against pathogens given its rapid response to non-specific antigens (56, 57). In this study, we measured three humoral parameters related to innate immunity: (a) RB activity, (b) hemolytic activity of the alternate complement system, and (c) plasma lysozyme concentration (**Figure 2**). Salmon held at 40% air sat. for six weeks had increased RB activity compared to fish maintained under normoxia (100% air sat.) by more than 50%. This finding is in contrast to data on Atlantic salmon exposed to intermittent hypoxia (3.5 times day^-1^ with a mean duration of 69 min) which showed a decrease in head kidney leukocyte RB (10), and on sablefish [*Anoplopoma fimbria* (29)] held at the same oxygen level as in this study where no effect of hypoxia was observed. However, this response is consistent with what Zanuzzo et al. (58) reported for Atlantic salmon chronically exposed to 60-70% air sat at 20°C. Collectively, these data suggest that hypoxia has different effects on this parameter depending on species and whether the period of oxygen deprivation is short-term (acute) or prolonged. Lysozyme concentration can be used as an indicator of the innate immune response (56, 59), since it is rapidly induced in fish exposed to stressors and has potent bacteriolytic activity (60). There is limited data on the effects of hypoxia on plasma lysozyme activity, although Ni et al. (61) did observe significant increases in total lysozyme protein levels after 6 h of acute hypoxia (5 mg L^-1^ and 3 mg L^-1^) in amur sturgeon (*Acipenser schrenckii*). In our study the increase in RB and lysozyme activity may have been primarily related to the increase in monocyte and granulocyte numbers as these cells produce both reactive oxygen species and lysozyme (62, 63). What resulted in the proliferation of these cells is not known. However, basal cortisol levels were much lower in this population of salmon when held under severe hypoxia vs. normoxia [i.e. ~ 8 vs. 16 ng mL^-1^; de Mello et al. (unpubl)], and this finding is consistent with Kvamme et al. (14) who also reported lower cortisol levels in hypoxia- vs. normoxia-acclimated Atlantic salmon when oxygen deprivation was prolonged. However, a difference in cortisol levels in normoxic vs. hypoxic sablefish was not observed in our previous study (29), and this finding might explain the disparity in results with regards to alternative complement activity and lysozyme concentration between the two species. Elevated cortisol levels have been reported to result in decreased lymphocyte counts and disease resistance in fishes (64, 65).

In contrast to the results for RB and lysozyme activity, we did not detect any difference in the activity of the alternate complement system (**Figure 2B**), which plays a crucial role in the host defense and is composed of a wide range of plasma proteins that lyse pathogens (66). However, this was not unexpected. Previous studies have reported that hypoxia does not change the activity of the alternate complement system. For example, both Magnoni et al. (67) and Leeuwis et al. (29) failed to detect any changes in alternative complement activity when rainbow trout (*Oncorhynchus mykiss*) and sablefish, respectively, were held in hypoxic conditions for prolonged periods. This may be because alternative complement is a basal/constitutive component of the defense against pathogens (i.e., is always active) (68). So much so that complement proteins are transferred from parents to progeny (eggs) (69).

Erythrocytes (RBC) are the predominant cells in circulation, and an increase in erythrocyte numbers is a typical response to the lack of oxygen as this will/may help to acquire more oxygen at the gills and optimize oxygen transport to the tissues (70). This was not observed in our fish, and suggests that at the temperature that the fish were held (10°C) blood oxygen transport capacity was sufficient to meet the Atlantic salmon’s basal requirements.

#### 4.1.2 Immune-related gene expression

To examine the effects of chronic hypoxia on the Atlantic salmon’s immune system at the molecular level, we measured the expression of eight immune-related genes in the head kidney, including *campb*, *ccl19b*, *cox2*, *hampa*, *il1b*, *il8a*, *saa5* and *strl5.* Overall, our results suggest that salmon held under conditions of chronic severe hypoxia have an enhanced constitutive (basal) innate immune capacity, as shown by the higher expression of biomarkers related to bacterial recognition (*stlr5*), the inflammatory process (*il8a*), antibacterial activities (*campb*, *hampa*) and lymphocyte recruitment/proliferation (*ccl19b*) (**Figure 4**). TLRs have traditionally been associated with pathogen recognition (71), however, these molecules can also recognize hypoxia-related molecules such as heat shock proteins (HSPs) and hypoxia inducible factors (HIFs). This has been demonstrated in human endothelial cells and murine macrophages (72, 73), and not surprisingly, prolonged hypoxia resulted in a significant upregulation of this recognition factor in catla (*Catla catla*) (74). This observation is also in accordance with our results, as basal (constitutive) *stlr5* expression was ~7-fold higher in hypoxia-acclimated salmon (**Figure 4H**), and liver *hsp47* (also known as *serpinh1*), but not *hif1ac* (*hypoxia inducible factor 1 alpha c*), transcript expression levels were significantly higher in salmon chronically exposed to 40% air saturation (de Mello et al., unpubl.). However, it is contrary to the data for hypoxic vs. normoxic sablefish, and thus, a response to lower levels of cortisol cannot be excluded for *stlr5* expression, or the transcript expression of any of the other genes that we report to be upregulated by chronic exposure to 40% hypoxia (see below). It has been proposed that TLR expression is also linked to the concomitant upregulation of some interleukins in channel catfish (*Ictalurus punctatus*) exposed to hypoxic conditions (75). Interleukins are known to act as pro-inflammatory cytokines that mediate immune responses (76). Although we did not see any changes in basal *il1b* expression, the basal expression of *il8a* was significantly upregulated (~2.7-fold change; **Figure 4F**) in the hypoxia-acclimated fish. Interleukin-8 is a chemokine that specifically attracts neutrophils and basophils (both granulocytes), and lymphocytes, to sites of infection (77, 78). Therefore, not surprisingly, the higher proportion of leukocytes in the hypoxia-acclimated fish is in accordance with an up-regulation of constitutive levels of *il8a*. In contrast, however, neither *il8a* expression nor leukocyte counts were different in sablefish acclimated to hypoxia (29), and again this likely reflects the much greater hypoxia tolerance, and the differential effects of hypoxia on the stress and physiology, of these fishes [(29, 79); de Mello et al., unpubl.].

The gene *ccl19b* is a proinflammatory chemokine that plays a central role in homeostasis and in the immune response (80), and is one of the most evolutionary conserved members of the chemokine family (81). Hypoxia-acclimated Atlantic salmon showed an upregulation of this gene under basal conditions (by ~1.6-fold; **Figure 4B**). Importantly, this chemokine is expressed and regulated by leukocytes [reviewed by (82)], and thus, its upregulation was not surprising given the higher leukocyte counts in the hypoxia-acclimated fish. In fishes, *ccl19* paralogues have been traditionally linked to the immune response to pathogens (81, 83), while other CC chemokines-like molecules have been related to physiological changes, including those associated with hypoxia (82). Interestingly, *ccl19* is negatively related to angiogenesis, a common mechanism of adaptation to low oxygen conditions (83, 84), but regulatory mechanisms need to be further investigated. Another mechanism of adaptation to hypoxia includes alterations in glycolysis [reviewed by (80)]. Despite this, we did not measure metabolic parameters in the present study, and it would be relevant to investigate possible interactions between adaptive mechanisms to low oxygen conditions in the Atlantic salmon and the trade-off with immune status.

Cathelicidin (CAMP) and hepcidin (HAMP) are cationic anti-microbial peptides (AMPs) which have been traditionally associated with the direct lysis of pathogens (85, 86), but also with immunomodulatory functions and inflammation (87–89). We report an upregulation of both *campb* and *hampa* (by 1.6- and 1.5-fold, respectively) basal expression in hypoxia-acclimated salmon (**Figures 4A, D**). HAMP activity is regulated by iron storage status and systemic inflammation, and is a major inducer of hepcidin expression (90). In humans, hypoxic conditions suppress HAMP activity, and hypoxia promotes erythropoiesis to enhance oxygen transport by inducing the production of erythroferrone, a suppressor of HAMP (91, 92). However, we observed the opposite. Hypoxia-acclimated salmon upregulated the expression of *hampa*, and furthermore, the proportion of immature erythrocytes did not differ between salmon under basal conditions. This suggests that either (1): these fish adapted to hypoxic conditions by lowering their metabolism and not by increasing oxygen uptake capacity; and/or (2) they are in a state of chronic inflammation, as showed by the increased leukocyte numbers and inflammatory biomarkers. On the other hand, CAMP can stimulate angiogenesis to increase oxygen delivery to tissues, and inhibit the inflammatory response by protecting the endoplasmic reticulum from stress (93–96). It is expressed in leukocytes (97), and thus, the increase in its expression is not surprising given the hypoxia-induced increase in these cells.

#### 4.1.3 Serum IgM levels

It is known that IgM levels depend on a number of factors including species, age, weight, gender, season, and infection and vaccination status [reviewed by (98)]. Previous studies have shown that IgM titers in Atlantic salmon are generally low as compared to other species like rainbow trout (9 mg mL^-1^), halibut (*Hippoglossus hippoglossus*, 4 mg mL^-1^) and Atlantic cod (*Gadus morhua*, 11.5 mg mL^-1^) (99, 100). However, we report serum IgM levels of ~13 to 16 mg mL^-1^ in hypoxia and normoxia, respectively; these values more than 10 times higher than reported by Magnadottir et al. (101) for 2-5 kg Atlantic salmon reared in land based tanks at 2-8°C (~1 mg mL^-1^). We cannot explain this discrepancy at this time. However, the important finding here is that prolonged hypoxia did not affect serum concentrations of IgM in our Atlantic salmon. Immunoglobulins enhance antigen-specific opsonization and phagocytosis, and are synthesized by lymphocytes (102), and thus, this finding is in agreement with the lack of a change in lymphocyte counts in this study and in pikeperch [*Sander luciopera* (30)] and sablefish (29) when exposed to hypoxia.

### 4.2 Hypoxia-related impacts on antigen-stimulated immune function

#### 4.2.1 Humoral immunity and blood cell populations

While pre-injection numbers (the percentage) of mature or immature RBCs did not differ between acclimation conditions in this study, the percentage of the former was higher in hypoxia-acclimated fish following the injection of both PBS and *Asal*, and an increase in immature erythrocytes following injection was only seen in this group (**Figures 3A, B**). These data suggest that chronic exposure to 40% air saturated water resulted in greater storage of these cells in the spleen, and/or a greater release of these cells. In support of this hypothesis, it has been reported for many fish species that stress causes splenic contractions and the release of RBCs into the circulation to deal with increased oxygen demands [e.g., in rainbow trout (*O. mykiss*) (103), tambaqui (*Colossoma macropomum*) (104) and amur sturgeon (*A. schrenckii*) (61)]. However, it is also possible that this increase in post-injection mature and immature RBC’s in the hypoxia-acclimated fish simply reflected the larger decrease in post-injection leukocyte numbers in this group (**Figures 3C, D**; see below). In contrast to the results of this study, there was no effect of this environmental challenge on sablefish post-injection (stress) blood erythrocyte numbers/percentages. The sablefish is an extremely hypoxia- tolerant fish (79), and it is possible that such increases in blood oxygen capacity are not needed in this species until hypoxia becomes more severe.

After both PBS and *Asal* injection, RB and lysozyme activity increased in fish held under normoxia, while no increase was observed in fish acclimated to hypoxia (**Figures 2A, C**). This suggests that the latter group was unable to increase these parameters in the blood after an acute stimulus (stressor). Again, changes in RB, lysozyme activity and the number/percentage of circulating leukocytes (especially granulocytes and monocytes) were similar in magnitude (**Figures 3C, D**), and thus, we hypothesize that these differences between the two groups were due to a reduction in leukocyte production/activation in fish kept under hypoxia, and not to the migration of white blood cells to the inflammation site. In this case, however (as opposed to under basal conditions), the injection of PBS and *Asal* did not result in a difference in plasma cortisol levels in the two groups (de Mello et al., unpubl.), and this strongly suggests that the reduction in leukocyte numbers, RB and lysozyme activity in hypoxia-acclimated fish was not stress-related, but directly linked to the lower oxygen levels to which these fish were exposed. Our results agree with data on Nile tilapia (*Oreochromis niloticus*) held at low (0.1-1.5 mg L^-1^), medium (3.0-3.5 mg L^-1^) and high (6.5-7.0 mg L^-1^) dissolved oxygen (DO) levels for 12 weeks, where fish injected with *Aeromonas hydrophila* had a reduced lysozyme concentration at the lower DO levels (17). On the contrary, Leeuwis et al. (29) reported no differences in lysozyme activity after sablefish exposed for 16 weeks to hypoxic conditions were injected *Asal*. Overall, their results suggest that chronic hypoxia may have little effect on the innate immune system in teleost species that are tolerant of low water oxygen levels.

Regardless of the injection (i.e., PBS or *Asal*), the post-injection activity of the alternative complement pathway was not different between the two groups (**Figure 2B**). Again, this finding contrasts with the results for sablefish, where the activity of this pathway decreased in hypoxia-acclimated sablefish injected with *Asal* (29).

#### 4.2.2 Immune-related gene expression

Phosphate buffered saline injection resulted in relatively minor increases in transcript expression at 6 or 24 hpi, however, *campb*, *cox2* and *il1b* expression were significantly higher at 24 hpi in hypoxia- as compared to normoxia-acclimated fish (by 2.1- to 2.8-fold, **Figure 4**). In contrast, *Asal* injection resulted in very large increases in transcript expression, and this is consistent with other studies that have used formalin-killed formulations of this pathogen (29, 105) or other bacterial antigens (58) to stimulate immune-related gene expression or other physiological responses in Atlantic salmon. Importantly, however, the expression of 6 of the 8 genes was significantly lower in the hypoxia-acclimated fish at 6 and/or 24 hpi as compared to normoxia-acclimated fish, and the two where differences were not significant (*ccl19b* and *hampa*) showed a similar reduction in expression after *Asal* injection at 24 hpi (**Figure 4**). These results clearly show that antigen-stimulated gene expression is suppressed in hypoxia-acclimated salmon, and suggest that these fish would be more susceptible to bacterial infections during prolonged periods of low water oxygen levels. However, the reason(s) for this reduced antigen-stimulated gene expression is/are not known. Post-injection cortisol levels were only marginally (and not significantly) elevated in these Atlantic salmon, and no difference in post-injection cortisol levels was found between hypoxia- and normoxia-acclimated fish 6 or 24 hpi with PBS or *Asal* (de Mello et al., unpubl.). Further, although some authors suggest that mounting an immune response to pathogens/antigens is an energy-intensive endeavor [e.g., see (106)], and thus that redirection of energy to maintain homeostasis in the face of a stressor may reduce that available to deal with a pathogen (107), Zanuzzo et al. (105) did not detect any difference in the rainbow trout’s oxygen consumption when injected with *Asal* vs. PBS and this agrees with recent work on the coral reef damselfish *Pomacentrus amboinensis* (108). Overall, these data suggest that any metabolic expenditures associated with the innate immune response are minor, and not likely to impact the immune response at varied water oxygen levels. Despite this, we show that *ccl19b* expression was reduced by approximately ~50% in fish held at 40% air sat., and this suggests a reduced inflammatory response and is consistent with the lower number/percentage of leukocytes.

In a previous study, expression of six of the eight genes (*il1b*, *campb*, *il8a*, *hampa*, *cox2* and *stlr5*) we studied were measured in Atlantic salmon kept at 20°C under normoxic and hypoxic (60-70% air sat.) conditions and then vaccinated against bacterial pathogens (58). While the addition of moderate hypoxia did not affect peak post-vaccination transcript expression in these fish, the data suggested that hypoxia increased the duration of elevated immune-relevant transcript expression when fish were exposed to high temperatures. This finding is in contrast to our study and that of Kvamme et al. (14) who showed that long-term hypoxia reduced or delayed the expression of these genes in the salmon head kidney when these fish were injected with poly (I:C) or a water-based *V. anguillarum* vaccine at 10°C. Collectively, these studies suggest that hypoxia’s effect on innate immune gene expression is temperature dependent. However, studies directly investigating this question do not support this hypothesis. For example, Wang et al. (23) investigated the effect of 14 days of intermittent hypoxia at two temperatures on the immune response of Nile tilapia (*O. niloticus*) vaccinated with *Streptococcus agalactiae*, and showed that *il-1β*, *tnf-α* and *ifn-γ* were strongly down-regulated in hypoxic fish independent of temperature. As with other data we report in this paper, the decrease in antigen-stimulated gene expression in hypoxia-acclimated salmon does not agree with our previous study on the sablefish at similar DO levels and temperature. In the sablefish, for the vast majority of genes studies (i.e., 15 out of 16), hypoxia had no effect or increased *Asal*-stimulated gene expression, with *tlr5b* the only gene showing a decrease in transcript expression.

#### 4.2.3 Serum IgM

Contrary to the results for innate gene expression, we did not see a significant increase in IgM serum titers, or a difference in IgM titers in hypoxia- vs. normoxia-acclimated salmon, following the two injections of *Asal* (initial and boost; **Figure 5**). The reason(s) for the lack of IgM increase following *Asal* injection is/are not known as this bacteria’s antigens clearly stimulated the innate immune system. However, it may be due to the post-injection sampling point chosen. Using a similar *Asal* preparation, Leeuwis et al. (29) reported an increase in serum IgM titers in normoxic-acclimated sablefish at 10 weeks post-injection (wpi), but not at six wpi. Thus, it is possible that we would have been able to detect differences in serum IgM levels both post-injection and following hypoxic acclimation [as in (29)] if the fish had been sampled a few weeks later at 10°C. However, it may also be that the adaptive immune system of hypoxia-tolerant and intolerant fish species responds differently to prolonged hypoxia. For example, the tilapia, like the sablefish, is a very hypoxia-tolerant fish species, and Gallage et al. (109) showed that serum antibody titer was significantly lower in vaccinated Nile tilapia kept at 55% air saturation as compared to under normoxic conditions.

#### 4.2.4 Conclusions and perspectives

In this experiment we showed that: 1) chronic severe hypoxia improves basal/constitutive humoral immunity and immune-related gene expression in the head kidney; and 2) that while this condition limits the capacity of the innate immune system to respond to bacterial antigens, it did not appear to affect the adaptive immune system as measured by serum IgM titers. These data add considerably to our understanding of how the immune system of Atlantic salmon, and potentially other salmonids, responds to prolonged periods of severe oxygen limitation. However, it is unclear how these changes would influence the ability of these fish to defend against live pathogens. This is because: of the varied responses indicated above; there are very few studies that have examined the effects of chronic hypoxia on disease resistance in this taxon [but see (13)]; to our knowledge no other studies have examined how both the innate and acquired immune system respond to pathogens when challenged at low oxygen levels; and chronic hypoxia may cause an imbalance in the microbiome of the fish, which could lead to an increase in the abundance of opportunistic pathogens (110). Further, based on the few studies that have been conducted [e.g., this study (29, 109)], it appears that the immune system of hypoxia-tolerant fishes (e.g., sablefish, tilapia, carp) may respond differently to pathogens encountered when oxygen levels are low. Clearly, this is an area that demands further study, as are: the mechanisms by which hypoxia depresses the fish’s immune system; and the interactive effects of temperature and hypoxia on fish immune function. Post-injection cortisol levels were not different in this population of Atlantic salmon (de Mello et al., unpubl.), and there is little evidence to support the belief that the immune system requires large metabolic expenses and must be curtailed when oxygen availability is limited. Thus, it is still unclear how hypoxia mediates its effects on the piscine immune system. Further, environmental effects on fish immune function were recently reviewed by Franke et al. (111), and the limited data do not provide many valuable insights into how the fish immune system (and potentially survival) will be impacted by simultaneous exposure to these two climate change-related environmental challenges. Such studies should be a priority as average ocean temperatures are rising (8), heat waves are becoming more frequent and severe (112, 113), and some believe that the greatest challenge to fishes in the current era of climate change is not high temperatures, but hypoxia (114).

## Data Availability

The original contributions presented in the study are included in the article/**Supplementary Material**. Further inquiries can be directed to the corresponding authors.
